# Atorvastatin Modulates the Efficacy of Electroporation and Calcium Electrochemotherapy

**DOI:** 10.3390/ijms222011245

**Published:** 2021-10-18

**Authors:** Wojciech Szlasa, Aleksander Kiełbik, Anna Szewczyk, Vitalij Novickij, Mounir Tarek, Zofia Łapińska, Jolanta Saczko, Julita Kulbacka, Nina Rembiałkowska

**Affiliations:** 1 Faculty of Medicine, Wroclaw Medical University, 50-367 Wroclaw, Poland; wojciech.szlasa@outlook.com; 2 Medical University Hospital, 50-556 Wroclaw, Poland; aleksander.kielbik@outlook.com; 3 Department of Molecular and Cellular Biology, Faculty of Pharmacy, Wroclaw Medical University, 50-556 Wroclaw, Poland; a.szewczyk@umw.edu.pl (A.S.); zofia.lapinska@student.umw.edu.pl (Z.Ł.); jolanta.saczko@umw.edu.pl (J.S.); julita.kulbacka@umw.edu.pl (J.K.); 4 Department of Animal Developmental Biology, Institute of Experimental Biology, University of Wroclaw, 50-335 Wroclaw, Poland; 5 Institute of High Magnetic Fields, Vilnius Gediminas Technical University, 03227 Vilnius, Lithuania; vitalij.novickij@vgtu.lt; 6 Université de Lorraine, CNRS, LPCT, F-54000 Nancy, France; mounir.tarek@univ-lorraine.fr

**Keywords:** atorvastatin, electrochemotherapy, electroporation, calcium, cholesterol, actin

## Abstract

Electroporation is influenced by the features of the targeted cell membranes, e.g., the cholesterol content and the surface tension of the membrane. The latter is eventually affected by the organization of actin fibers. Atorvastatin is a statin known to influence both the cholesterol content and the organization of actin. This work analyzes the effects of the latter on the efficacy of electroporation of cancer cells. In addition, herein, electroporation was combined with calcium chloride (CaEP) to assess as well the effects of the statin on the efficacy of electrochemotherapy. Cholesterol-rich cell lines MDA-MB231, DU 145, and A375 underwent (1) 48 h preincubation or (2) direct treatment with 50 nM atorvastatin. We studied the impact of the statin on cholesterol and actin fiber organization and analyzed the cells’ membrane permeability. The viability of cells subjected to PEF (pulsed electric field) treatments and CaEP with 5 mM CaCl_2_ was examined. Finally, to assess the safety of the therapy, we analyzed the N-and E-cadherin localization using confocal laser microscopy. The results of our investigation revealed that depending on the cell line, atorvastatin preincubation decreases the total cholesterol in the steroidogenic cells and induces reorganization of actin nearby the cell membrane. Under low voltage PEFs, actin reorganization is responsible for the increase in the electroporation threshold. However, when subject to high voltage PEF, the lipid composition of the cell membrane becomes the regulatory factor. Namely, preincubation with atorvastatin reduces the cytotoxic effect of low voltage pulses and enhances the cytotoxicity and cellular changes induced by high voltage pulses. The study confirms that the surface tension regulates of membrane permeability under low voltage PEF treatment. Accordingly, to reduce the unfavorable effects of preincubation with atorvastatin, electroporation of steroidogenic cells should be performed at high voltage and combined with a calcium supply.

## 1. Introduction

Pulsed electric field (PEF) may induce various effects when applied to cells, for instance affecting the integrity of their plasmic membranes. Electroporation (EP) refers to the phenomenon describing the permeabilization of cell membranes subjected to the electric fields of intensity well above a threshold, believed to result from the formation of pores. Such pores allow the release or intracellular material or the uptake of drugs or genetic material by the cells [1,2,3] in electroporation-based technologies and treatments. In clinical setups where electroporation is combined with chemotherapeutics, the method is known as electrochemotherapy (ECT), and calcium electroporation (CaEP) is a specific subset in which calcium ions are the substance to deliver to treated cells. In CaEP, a high concentration of calcium is introduced to the target cancer cells and the technique is therefore used in anti-cancer therapy [4,5]. The advantages of CaEP and the growing interest in this chemical compound instead of traditional cytostatics, e.g., bleomycin, are the low systemic toxicity of the therapy, its applicability in small mobile outreach clinics, and of course the very low cost of the “drug” [4]. With appropriate electric pulse parameters, the cells can restore their compromised membrane integrity. In this case, the technique used is known as reversible electroporation and it is applied as a non-viral transfection method or for targeted delivery of hydrophilic drugs to the cells [6]. The cell resealing mechanisms are complex and strongly dependent on the membrane proteins [7] and lipid content [8], the membrane fluidity [9], and external factors such as temperature [10].

Most of the research aiming to increase the efficiency of ECT concerns optimization of the electroporation’s protocols [6,11,12,13,14,15]. Alternatively, enhancing the effect of CaEP might be achieved by modifications of the physical properties of the targeted cells to make them more susceptible to EP. Hence, factors modulating the cell membrane permeability such as cell membrane lipid content, can be considered a target of interest [12].

One of the most important lipids in cellular membranes is cholesterol [16], the concentration of which regulates the cell membrane fluidity and permeability [17,18]. Moreover, its metabolism correlates with the progression of the tumor [19]. Cholesterol plays a chemoprotective role in lipid membranes reducing their oxidation [20]. In cancer cells, its high content confers higher resistance to chemotherapy [21].

Another factor that regulates cell membranes’ permeability is the organization of their actin fibers network, as shown by Muralidharan et al. [22]. Via the zyxin-mediated connection with the cell membrane, actin fibers regulate the surface tension of the cell membrane. Therefore, changes in the organization of actin might influence the permeability of the cell.

Statins are commonly used drugs for hyperlipidemia that provide remarkable benefits to patients with risks of coronary heart disease or type 2 diabetes mellitus [23]. Due to their proven efficiency and high-safety profile, statins found use as first line lipid-altering therapy. Statins act by inhibiting 3-hydroxy-3-methylglutaryl-coenzyme A (HMG-CoA) reductase and, accordingly, also inhibit the production of cholesterol [24]. Furthermore, statins exhibit anti-cancer activities [25]. Among statins, atorvastatin stands out given its clinical effectiveness and favorable pharmacokinetics, such as high solubility and permeability through the cell membrane [26,27]. Fan et al., in 2016 proved that atorvastatin impedes the formation of actin stress fibers and prevents the downregulation of E-cadherin [28]. Therefore, the application of atorvastatin would potentially modulate both the permeability of the cell membrane towards PEFs as well as the metastatic potential of the cell.

PEF treatment and the dysregulation of cholesterol cellular homeostasis by the stimulation with atorvastatin may also influence the expression and localization of membrane proteins. One of the most serious outcomes might be the increase of the metastatic potential of the cancer cells. Therefore, the examination of proteins that play a significant role in oncogenesis and cancer progression is crucial in studies concerning the modulation of cells’ fluidity [23]. N-cadherin is an example of raft-bound cell contact protein. It is expressed on mesenchymal cells [26,27,28]. The transition in the membrane expression of E- to N-cadherin results in the epithelial to mesenchymal transition and correlates with metastatic properties of the carcinomas [24,29,30].

In this study, we carried out a range of biophysical and biochemical experiments to evaluate the effects of atorvastatin on cancer cells when administered in combination with electroporation and calcium electrochemotherapy. First, we determined the effects of statin on the cholesterol content and relative zyxin-actin organization. Subsequently, we studied the effects of atorvastatin on membrane permeability. All the data were supported with molecular dynamics modeling. Further experiments were conducted to assess the cytotoxic effects induced in cancer cells by incubation with atorvastatin followed by EP and CaEP. In the end, we verified if statin modulates the metastatic or adhesion markers of the cell by tracking the expression of E- and N-cadherins. We proposed a model in which actin and cholesterol simultaneously regulate the permeability of the cell membrane.

## 2. Results

### 2.1. Effects of Atorvastatin Preincubation on Cholesterol Content and Actin Organization

#### 2.1.1. Cellular Cholesterol Quantification and Distribution after Preincubation with Atorvastatin

**Figure 1:**
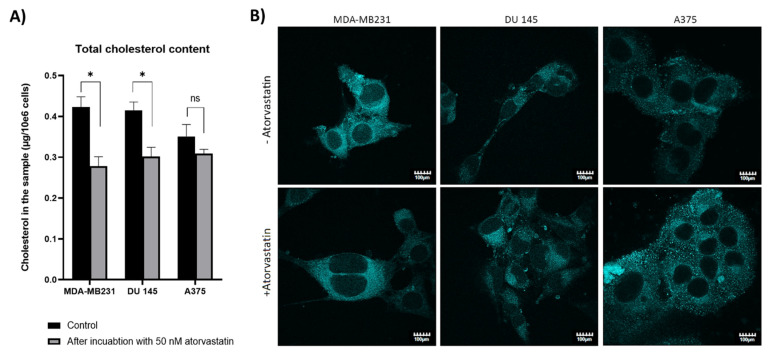
Cholesterol distribution and content studies. (**A**) Changes in the cholesterol content in MDA-MB231, DU 145, and A375 cells incubated for 48 h with 50 nM atorvastatin. Data presented as mean ± SD, * *p* < 0.05 and ns—not significant difference (*n* = 3); (**B**) distribution of cholesterol the MDA-MB231, DU 145, and A375 cells treated with 50 nM atorvastatin for 48 h.

Figure 1A reports the changes in the amount of cholesterol in cells preincubated with atorvastatin. The data indicate that overall, the preincubation decreases cholesterol levels in the cells, yet with differences depending on the cell line. The statistical difference between the preincubated cells and the control ones was the highest in MDA-MB231 (~ 0.15 μg/10^6^ cells) and in a similar range for the DU 145. The effect of statin preincubation was the lowest in the A375 cells, which induced a decrease of the cholesterol content by merely 0.05 μg/10^6^ cells, a change that is not statistically significant.

The distribution of cholesterol in the cells that underwent the same preincubation treatment, depicted from confocal microscopy shown in Figure 1B, indicated that each cell line presents a similar pattern. For MDA-MB231, the fluorescence signal of control cells seemed even, and after treatment with 50 nM atorvastatin, it emanated mainly from the intracellular compartment. In the case of DU 145 cells, the initial fluorescence seemed to be even, and after preincubation with 50 nM atorvastatin, the signal was clearly higher near the nucleus. In general, A375 cells showed a more granular pattern of cholesterol distribution; however, the tendency was still retained.

#### 2.1.2. Effect of Atorvastatin on the Actin System of the Cell

**Figure 2:**
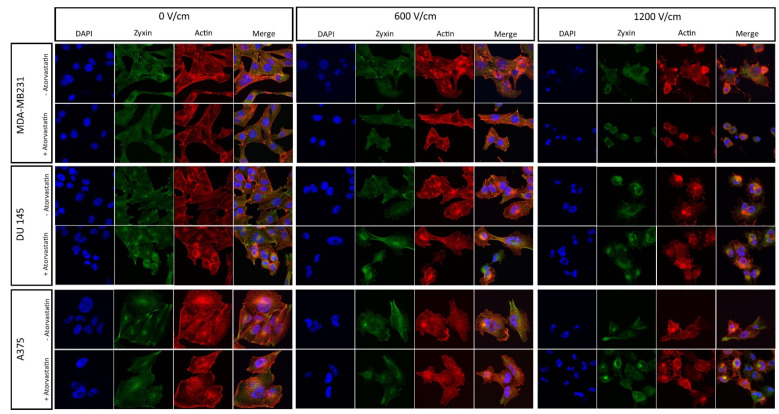
Actin, zyxin, and nuclei organization in MDA-MB231, DU 145, and A375 cells after 48 h incubation with 50 nM atorvastatin followed by electroporation. Six hours after the experiment, the cells were stained and observed on the confocal laser microscope, 60× magnification.

Figure 2 reports the structural changes in the actin organization and zyxin distribution of the three cell lines after 48 h incubation with 50 nM atorvastatin. Properly organized actin showed fibrils rather than cytoplasmic globular structures. In the case of normal zyxin, it was organized in the periphery of the cell, in the places in which actin fibers ended. There was no extensive cytoplasmic expression of the protein.

For the MDA-MB231 cells, the changes in zyxin and actin were subtle. When treated with atorvastatin or subjected to low voltage PEF (600 V/cm), there was nearly no effect of the statin on actin and zyxin. The difference in cells’ morphology might be observed when subjected to 1.2 kV/cm PEF treatment. Namely, the cells preincubated with the statin shrink, and no regularities in actin nor zyxin organization were observed. The control sample was highly affected, but the cells remained less disorganized, and the structural components of the cells were retained.

The actin system of DU 145 prostate cancer cells preincubated with the statin appeared to shift from the stress-fibers system to the globular structures, irregularly aligned in the cytoplasm. Furthermore, the standalone statin treatment induced the disorganization of zyxin towards cytoplasmic diffused distribution in contrast to peripherally aligned control cells. The effect was exacerbated with the increase in PEF intensity.

Standalone treatment with atorvastatin induced noticeable changes in the A375 melanoma cells, which shifted from the fibrillary actin to globule-like structures. In case of zyxin, it might be observed in the periphery of the cell in samples not subjected to PEF treatment. However, when 600 V/cm PEF was applied, both zyxin and actin became disorganized. Namely, zyxin shifted towards the cytoplasmic, diffused expression and actin shows merely fibrillary structures. Most actin fibers were organized in the irregularly shaped structures in the cytoplasm, and the remaining fibrils remained not parallel with respect to each other. The effect was more visible in the cells preincubated with atorvastatin. Conversely, when subjected to 1.2 kV/cm PEF, A375 cells and cells preincubated with atorvastatin were both destroyed and it was hard to observe any differences.

### 2.2. Cell Membrane Permeabilization

**Figure 3:**
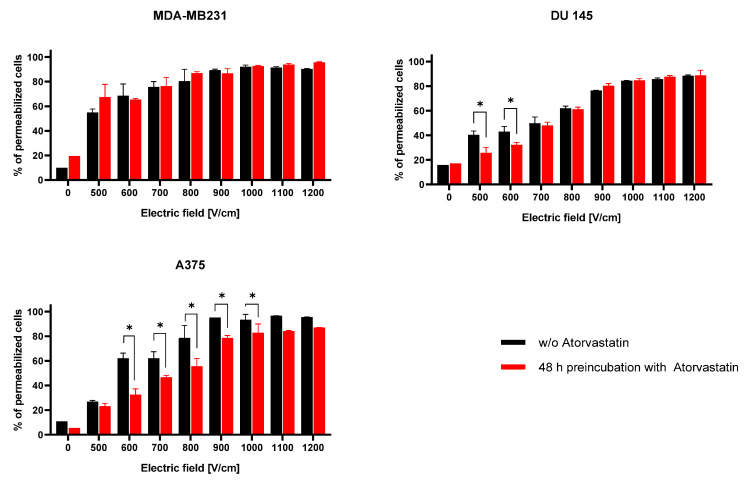
The uptake of Yo-Pro-1 after treatment with PEF ranging from 500 to 1200 V/cm (8 pulses, 1 Hz). The three plots present different cell lines: MDA-MB231, DU 145, and A375. Within one cell line, one culture was treated with 50 nM atorvastatin for 48 h and the other was the control. Cells were treated with PEFs (0, 500–1200 V/cm, 8 pulses, 0.1 ms pulse duration) in the buffer with Yo-Pro-1. Data presented as mean ± SD, * *p* < 0.05.

To assess if the changes in the cells’ cholesterol content influence their permeability when subjected to electric field pulses, we used Yo-Pro-1 dye uptake protocol. We compared each cell line’s results for cells preincubated with atorvastatin for 48 h to non-exposed control samples. Figure 3 reports the dye uptake following PEF treatments of intensities ranging from 500 to 1200 V/cm. As expected, the largest cells (MDA-MB231) were characterized by higher Yo-Pro-1 uptake in PEFs of 500 V/cm and were easier to permeabilize in lower electric fields than the other cell lines. A375 and DU 145 cells after preincubation with atorvastatin were less permeable to Yo-Pro-1 than the cells subject only to electric fields. For the DU 145 cell line, the effect was particularly noticeable in the low PEF intensity range (500–700 V/cm), while for A375 line, the phenomenon was observed across the wide range of PEF amplitudes.

### 2.3. Molecular Dynamics Studies of the EP Threshold as a Function of Cholesterol Content

Molecular dynamics studies were performed to shed light on the mechanisms of electroporation regulation. We investigated how cholesterol content in simple cell membrane models affects the response to PEFs of low and high intensities.

We first, for each system composition, determined the electroporation threshold, i.e., the voltage under which pores form in the bilayers. Further, to characterize the role of surface tension, we analyzed the effects of positive and negative lateral membrane tensions as well. Actin is indeed connected to the cell membrane via zyxin, thus also involved in regulating cell shape and presumably the increased organization of the fibers leading potentially to modulation of the cell membrane surface tension might affect the electroporation efficacy as indeed, a change in the membrane surface tension would affect the electroporation threshold (potential required to form pores).

**Figure 4:**
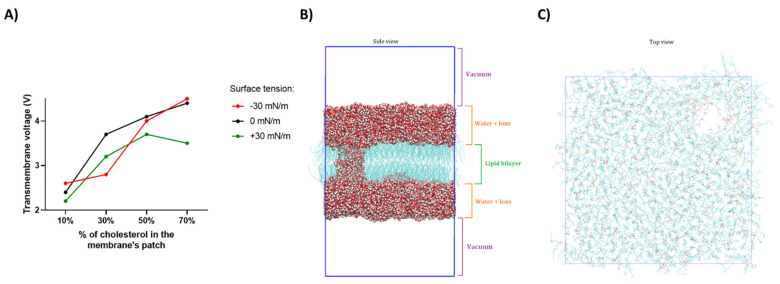
Membrane’s patch electroporation threshold after application of μsPEF with respect to the cholesterol content-molecular dynamics studies. (**A**) The graph shows the minimal ionic imbalance across the simulated membrane, which is needed to form a pore in the membrane; (**B**) shows the electropore throughout the membrane induced by the ionic imbalance in the system composed of two water slabs separated by the membrane and vacuum; (**C**) shows the top view of the electropore in the membrane.

Figure 4 reports data related to the permeability of the membrane models at different cholesterol contents when subject to μsPEF. The molecular dynamics simulations show that a higher voltage must be applied to electropore membranes with increasing cholesterol content. Interestingly, in the membrane patches with 30% cholesterol, the decrease in the surface tension significantly decreased the electroporation threshold. As for the positive surface tension, it decreased the electroporation threshold in the whole range of the analyzed cholesterol content. Curiously, in cholesterol-rich patches, the EP threshold of the membrane under 0 surface tension remained similar to that of negative surface tension. Conversely, cholesterol-poor patches under 0 surface tension responded to PEFs in a similar way to the patches under the positive surface tension value.

Knowing that pores are likely to form in the regions where the threshold is the easiest to exceed, we may assume that in physiological conditions, the cell membrane is electroporated in cholesterol-low regions, and with the increase in the surface tension, the electroporation threshold decreases.

Interestingly, molecular dynamics studies are consistent with the actin/zyxin staining and Yo-Pro-1 uptake results. Namely, when the actin fibers are disrupted by atorvastatin, the surface tension of the cell membrane decreases. In this case, according to our MD studies, in the regions where the cell membrane is electroporated, the electroporation threshold should increase and indeed it did. Conversely, when the actin fibers are disrupted by the high-voltage electric field (1200 V/cm), there is mainly no difference between the cells preincubated with atorvastatin and the ones without the preincubation.

### 2.4. Viability Assays

#### 2.4.1. SRB Assay

**Figure 5:**
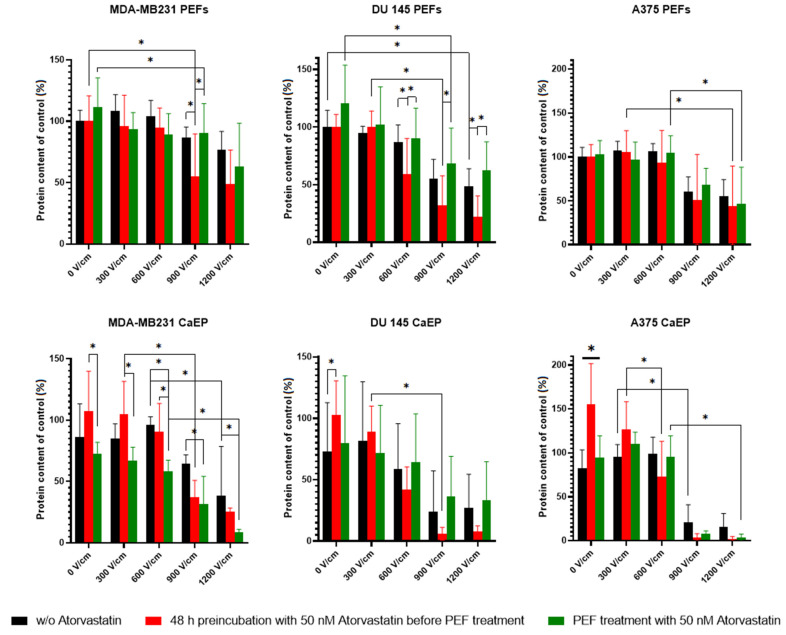
SRB assay. The total protein content in the cells measured 72 h after PEF with and without 5 mM calcium ions on MDA-MB231, DU 145, and A375 cell lines. Black bars: cells without atorvastatin; red bars: cells preincubated with 50 nM atorvastatin for 48 h before PEF treatment (long-term effects); green bars: the cells which were treated with 50 nM atorvastatin directly in the electroporation buffer (short-term effects). All the data were calculated as the % of untreated cells, i.e., without 0 V/cm PEF and without the addition of calcium ions. Data presented as mean ± SD, * *p* < 0.05 (15 > *n* > 5). On average, the cells preincubated with 50 nM atorvastatin reacted differently with low and high voltage PEFs. We observed that the response of the cells was different to the therapy, depending on the applied electric field intensity. Namely, in low voltages, the total protein in the cells preincubated with atorvastatin remained increased and with the elevating electric field intensity, the total protein content decreased in comparison to control cells.

To examine the total protein content in the cells submitted to PEFs, we performed a Sulforhodamine B (SRB) assay. SRB binds stoichiometrically to proteins under mildly acidic conditions, and thus the assay measures the total content of proteins in the cell and can be interpreted as the structural and functional activity of the cell. To assess the impact of atorvastatin administration, we analyzed both cells preincubated with 50 nM statin for 48 h before PEF treatment (long-term effects) and cells subjected to PEFs with 50 nM atorvastatin added to the electroporation buffer (short-term effects). Control cells treated with standalone PEFs were studied as well. All three of the mentioned protocols were carried out with and without the addition of 5 mM calcium chloride, allowing, therefore to investigate both EP and CaEP. Figure 5 presents the results of the SRB assay conducted 72 h after the PEF treatments.

Concerning the standalone PEFs treatment, MDA-MB231 and DU 145 cells preincubated with 50 nM atorvastatin and treated with PEFs of intensity above 600 V/cm presented less protein content than the control cells. For A375 melanoma cells, this effect was not observed. The addition of the same amount of atorvastatin, namely 50 nM, to the electroporation HEPES buffer before PEF treatment did not lead to specific changes as compared to the control. For the A375 melanoma cell line, both PEFs treatment protocols with atorvastatin had a similar effect and noticeably, the cell viability recorded after PEFs treatments with intensities ~900 V/cm or greater was about null 0%. In fact, the cytotoxic effect (decrease of protein content) among breast and prostate cancer cells was significantly lower than in the statin-preincubated cells.

In the set of PEFs treatment in the presence of calcium, the results of the essay were quite different. For the MDA-MB231 breast cancer cell line, the direct addition of atorvastatin to the electroporation buffer led to a higher decrease in protein content compared to that measured for preincubated samples. In the case of melanoma and prostate cancer cells, the mentioned tendency was not observed.

#### 2.4.2. MTT Tetrazole Assay

**Figure 6:**
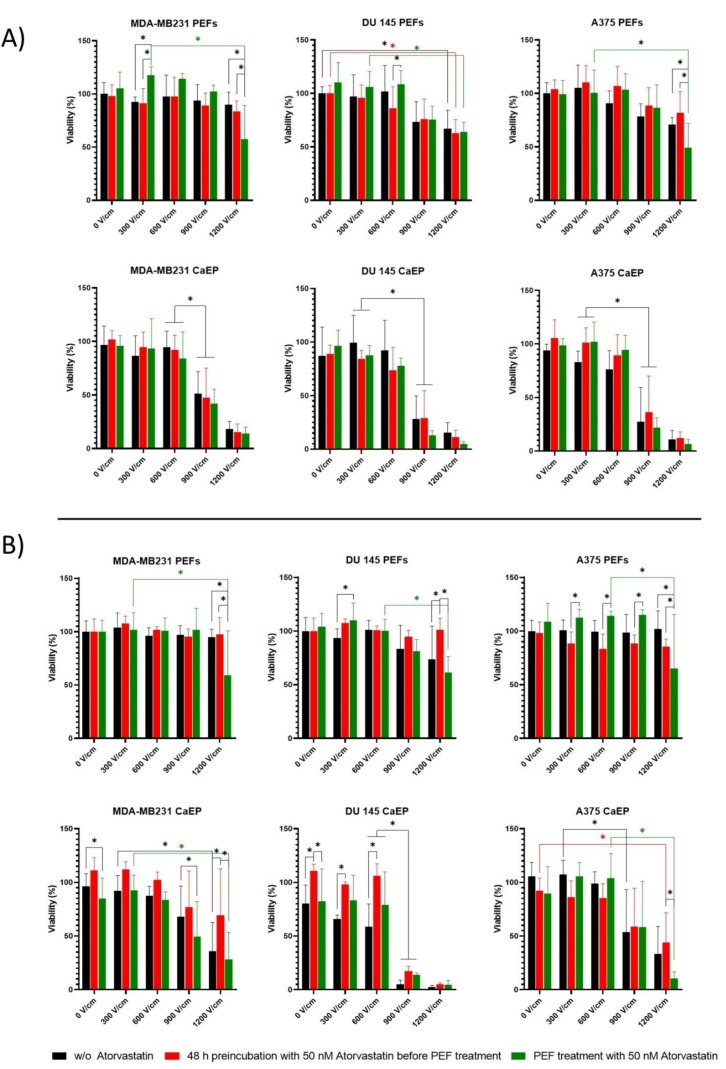
MTT assay. Cells’ viability expressed by the mitochondrial activity of the cells measured. (**A**) 48 h after PEF with and without 5 mM calcium ions on MDA-MB231, DU 145, and A375 cell lines. Data presented as mean ± SD (21 > *n* > 6); (**B**) 120 h after PEF with and without 5 mM calcium ions on MDA-MB231, DU 145, and A375 cell lines. Data presented as mean ± SD, (17 > *n* > 6). Black line: cells without atorvastatin; red line: cells preincubated with 50 nM atorvastatin for 48 h before PEF treatment (long-term effects); green line: the cells which were treated with 50 nM atorvastatin directly in the electroporation buffer (short-term effects—without 48 h preincubation time). ANOVA analysis * *p* < 0.05.

To investigate how addition of atorvastatin affects the cytotoxic potential of ECT alone, cell viability studies of the three cancer cell lines were conducted using the MTT assay (cf. Methods). Figure 6A shows the effects of PEFs on atorvastatin preincubated cells. In the case of electroporation cell viability measured within 48 h of the PEF treatment, there were no differences between the tested protocols except the one that involved the 1200 V/cm PEF treatment with 50 nM atorvastatin. In the case of CaEP, there were nearly no differences between the analyzed protocols.

Figure 6B shows the long-term (120 h) effects of the combination of PEF with atorvastatin on the cells’ mitochondrial activity. The extended incubation time was examined to assess if the alterations in the cell’s viability change over time. In the case of treatment without calcium, the preincubation with atorvastatin did not lead to any significant changes compared to control samples. However, for both MDA-MB231 and A375 cells, PEF treatment with 50 nM atorvastatin led to a high viability decrease when a 1200 V/cm field was applied. In contrast, with 5 mM calcium chloride, the MDA-MB231 and DU 145 cells subject to low voltage PEF treatment were more viable than control. Similarly, PEF treatment with 50 nM atorvastatin led to a high decrease in the cells’ viability when high voltages were applied.

The viability of cells exposed to PEF and preincubated or not with 50 nM atorvastatin was similar. However, in this case, the high efficacy of electroporation with the direct addition of atorvastatin to the electroporation buffer (1200 V/cm) was observed.

#### 2.4.3. Clonogenic Assay

**Figure 7:**
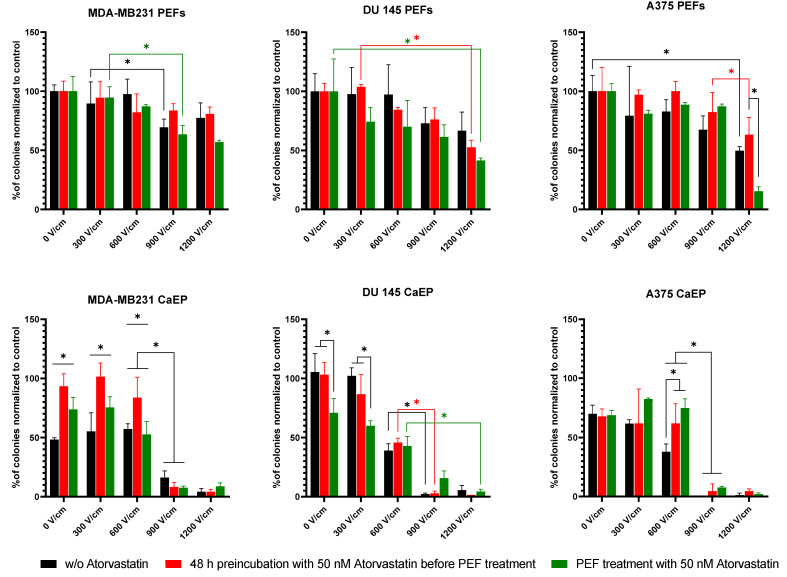
Colony-forming properties of the MDA-MB231, DU 145, and A375 cells after preincubation with 50 nM atorvastatin and electroporated with and without 5 mM calcium ions. To show the long- and short-term effect of the statin on the efficacy of PEF treatment, we analyzed three types of cells: Black line: cells without atorvastatin; red line: cells preincubated with 50 nM atorvastatin for 48 h before PEF treatment (long-term effects); green line: the cells which were treated with 50 nM atorvastatin directly in the electroporation buffer (short-term effects—without 48 h preincubation time). Data presented as mean ± SD, ANOVA analysis * *p* < 0.05.

The clonogenic assay enables an assessment of differences in the cells’ ability to produce progeny after treatment with different drugs or physical therapies [29]. Figure 7 shows the cells’ colony-forming properties after preincubation with atorvastatin and PEF treated with and without 5 mM calcium ions. Atorvastatin preincubation of MDA-MB231 cells had no effect when cells were PEF-treated in the absence of calcium ions. DU 145 prostate cancer cells were characterized with high susceptibility to electroporation in the presence of 50 nM atorvastatin. Interestingly, this was not observed for cells preincubated with the statin. When electroporated with 50 nM atorvastatin and 5 mM calcium chloride, the clone-forming decrease was less pronounced than for the control. For A375 melanoma cells, the electroporation without 5 mM calcium was the most effective when the cells were electroporated with 50 nM atorvastatin using the 1200 V/cm PEF. In the case of 5 mM calcium EP, the control cells formed more colonies than the preincubated or electroporated with 50 nM atorvastatin.

### 2.5. E- and N-Cadherins Confocal Microscopy Studies

**Figure 8:**
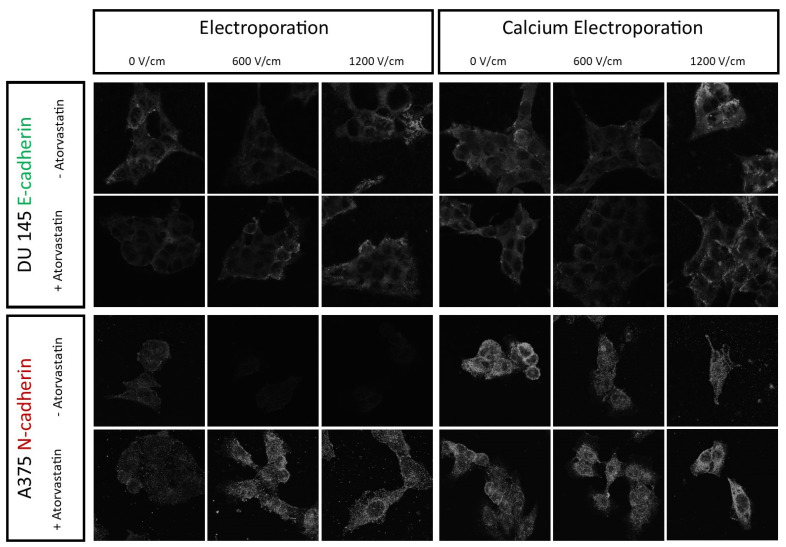
Confocal laser microscopy studies of E- and N-cadherins expression in the DU 145 and A375 cells after the atorvastatin-aided 5 mM calcium ECT. The layout is divided vertically into two sections—PEF treatment and CaEP. Further, each of them is vertically divided into three PEF intensities (0, 600, and 1200 V/cm). Each cell line is grouped and within each, the higher row shows cells not treated with atorvastatin and the lower one shows the cells preincubated for 48 h with the drug. Cells within one parameter were separately labeled with first order anti-N-cadherin or anti-E-cadherin antibody and then with second order Alexa 488 conjugated antibody. Samples were observed under Olympus F1000 confocal laser microscope, oil immersion lens with 60× magnification, NA: 1.35 was used to observe the samples.

N-cadherin immunofluorescence recorded on A375 cell showed that with the increasing PEF intensity, the localization of the N-cadherin’s signal shifts from the cell membrane to the cytoplasm of the cell (Figure 8). Depending on the conditions of PEF treatment, the signal of N-cadherin varied, i.e., when the cells were treated with the standalone PEF, N-cadherin signal decreased with the increasing intensity of the electric field. Conversely, N-cadherin’s signal expanded with the increase in PEF intensity when the cells were subjected to standalone PEF treatment after the incubation with atorvastatin. In the case of calcium electroporation, the signal remained high, but under high-voltage PEF treatment, the signal was localized mainly in the intracellular compartments, rather than in the cell membrane.

A similar tendency was observed with E-cadherin on DU 145 cells. Namely, 600 V/cm PEF induced the shift of the signal from the periphery of the cell, to the even cytoplasmic localization. Moreover, the fluorescence decreased, which might be due to the signal dissipation on the larger area. Curiously, after treatment with 1200 V/cm PEF, the signal of E-cadherin gathered in sphere-like structures, which are probably the aggregates of the protein in the dying cells.

## 3. Discussion

Given the high potency of electroporation as a method to induce cancer cells’ death and the rising number of clinical applications of electrochemotherapy [4,15,30,31,32,33,34,35], we felt it is important to characterize the effects among others of widely used drugs on its efficacy [36,37,38,39,40]. This paper analyzes how atorvastatin modulates CaEP and whether it can be used as a standalone electrochemotherapy agent. The data presented in this study were collected to investigate indeed how three cancer cell lines behave after exposition to the statin. First, we investigated how cancer cells respond to 48 h incubation with 50 nM atorvastatin. Both changes in the cholesterol content and the organization of actin fibers were assessed. Further, we performed cell membrane permeabilization studies and compared the effects on cells that underwent 48 h preincubation with atorvastatin with control cells. We performed viability experiments to analyze the effects of EP and CaEP on the total protein content (SRB), mitochondrial activity (MTT), and finally on the progeny forming properties of the cells (clonogenic assay). Using atomistic modeling, we compared the effect of cholesterol content, both on the model membrane’s surface tension and on the regulation of the permeability induced by PEFs.

**Figure 9:**
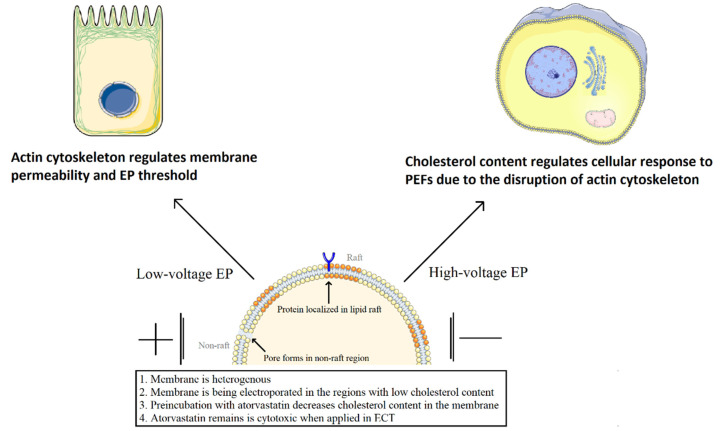
Mechanism of atorvastatin-aided EP.

Based on our experiments, we propose a mechanism in which cells react to the atorvastatin-aided ECT (Figure 9). The role played by actin in cell membranes’ resistance to PEFs was very recently discussed by Muralidharan et al. [22]. It was already proven that the membrane’s surface tension regulates the electroporation process in in vitro studies and simulations [2,41]. Tarek et al., provided evidence that the electric field induces significant lateral stress on the bilayer, manifested by surface tensions of magnitudes in the order of 1 mN/m [42]. Actin via zyxin participates in maintaining a surface tension, and if it is directly perturbed/damaged by electric fields, then the electroporation threshold should decrease. Combining all the current knowledge and the results from our experiments, we can propose a mechanism in which actin regulates the electroporation threshold. Namely, when actin reorganizes, the cell’s stretching resistance becomes enhanced. Graybill et al., showed the strong response of actin fibers towards the externally applied PEFs. This review also explained the cytoskeletal-related mechanism of cells’ response towards electric field [43].

Actin is connected to the cell membrane and acts as stress fibers preventing the cell from losing its shape. Thus, any change in the actin’s organization would result in altered resistance of the cell towards lateral membrane expansion. PEF treatment influences the cell’s shape as recently reviewed by Kotnik et al. [2] and critical changes in the morphology of the cells are induced following electroporation as reported by Rembiałkowska et al. [11].

Concluding, when the cytoskeleton is present it is responsible for maintaining the proper surface tension and regulating membrane permeability when subject to PEFs; however, the lipid composition of the cell membrane remains the regulatory factor of the membrane’s permeability when subject to actin-disrupting PEFs [44,45,46].

We have demonstrated that, when exposed to atorvastatin, typical steroidogenic cells (MDA-MB231 and DU 145) respond mostly by decreasing cholesterol content, an effect that is more pronounced for the former. In contrast, A375 melanoma cells respond mostly in a protein-related pathway by reorganizing actin fibers. In several previous studies, we showed that the reorganization of actin has a significant effect on the therapeutic outcome of the therapy of melanoma and prostate cancer cells and thus can be used as a target for the anti-cancer therapy [47]. We also showed that CaEP highly affects prostate cancer cells’ actin cytoskeleton [3,35].

To assess if the molecular changes induced by preincubation with atorvastatin alter the effects of EP and CaEP on the three cell lines, viability and clonogenic assays were performed. Overall, we found no differences between the control and cells’ viability preincubated with the statin when subject to PEFs. Curiously, the cells directly electroporated with 50 nM atorvastatin were significantly less viable when 1200 V/cm PEFs were applied, indicating great standalone toxicity of the drug. When 5 mM calcium was supplied, differences between the control and statin preincubation of DU 145 cells exposed to PEFs were found. The changes might arise from atorvastatin’s dualistic effect on both actin and the total cholesterol content, leading to higher resistance of the cells to low voltages. Concerning the cells’ colony-forming properties, only melanoma cells formed fewer colonies after direct electroporation with 50 nM atorvastatin. However, with a 5 mM calcium ions supply, a different effect on each cell line was noticed. The number of MDA-MB231 cancer cells colonies increased in response to preincubation with atorvastatin and 5 mM calcium chloride low voltage PEFs. A375 cells formed more colonies when electroporated directly with 50 nM atorvastatin and 5 mM calcium ions with low voltage PEFs. This observation is consistent with the high cytotoxicity of statins on melanoma cells generally reported [28]. Conversely, the DU 145 cells formed statistically fewer colonies after low voltage 5 mM CaEP with 50 nM atorvastatin.

Noticeably, the decrease of viability and colony-forming as a function of increased PEF intensities was always steepest for the atorvastatin preincubated cells. Namely, for the latter, the cells were more resistant to low voltage PEFs and less to high voltage PEFs. We observed no differences between the samples preincubated or electroporated with atorvastatin under 1200 V/cm PEF. These results imply that the effects of atorvastatin are observed above low-voltage PEFs, which are more dependent on the actin organization. However, due to the steepest loss of viability, the high voltage PEFs effects might be more related to the cell’s cholesterol content.

**Figure 10:**
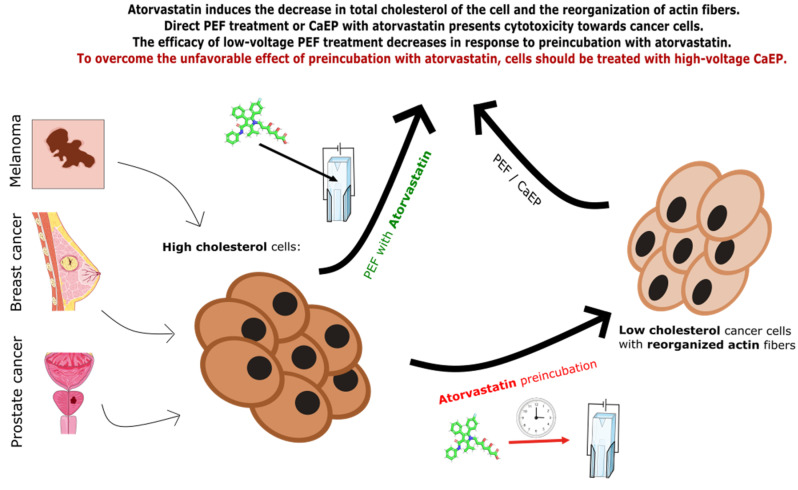
Study setup with the outcomes.

Statins were proved to modulate the expression of E- and N-cadherin in cell cultures [28,48,49]. For most of the patients on statin therapy, the drug is crucial to extend the life and increase the quality of life, so it is not possible to totally omit the use of atorvastatin during the course of therapy. In the case of E-cadherin expression, treatment of the cells with 1200 V/cm PEF (mostly used in ESOPE clinical protocol) did not affect the expression of the marker rather than its aggregation. However, the treatment of cells with the decreased PEF intensity (600 V/cm) led to the shift of both cadherins’ signal to the cytoplasm. Decrease of contact molecules from the cell membrane might be unsafe in the case of therapeutical applications. Our results show that it is possible to overcome the unfavorable effect of increased membrane N-cadherin expression by the use of high voltage pulses and combination with calcium supply. By doing so, the cells do not increase their membrane expression of N-cadherin. Therapy in the proposed protocol seems to be the safest choice when considering the simultaneous application of atorvastatin. Figure 10 summarizes the whole set of the experiments and the most important outcomes of the study.

Even though the study was extensive, there is still a need to conduct further in vivo animal studies to validate ours in vitro results. We hope the cell culture studies may highlight the problem of cholesterol modifier usage among patients undergoing ECT, and later, animal studies could be used to optimize ECT protocols in clinical practice. The authors believe that the problem should be more widely discussed and investigated in the future.

## 4. Materials and Methods

### 4.1. Cell Culture

For the study, three human cancer cell lines were used: MDA-MB231, DU 145, and A375 (ATCC, London, UK). A375 is a melanotic melanoma cell line, derived from the skin tissue of a 53-year-old male Caucasian patient. The MDA-MB231 breast adenocarcinoma cell line was obtained from the metastatic site—the pleural effusion of a 51-year-old Caucasian female adult. The DU 145 prostate cancer cell line was obtained from the metastatic site in the brain of a 69-year-old Caucasian male adult. Cells were grown in monolayer cultured in Dulbecco’s modified Eagle’s medium (DMEM, Sigma-Aldrich, St. Louis, MO, USA) supplemented with 10% fetal bovine serum (FBS, Sigma-Aldrich, St. Louis, MO, USA) and 1% of antibiotics solution (penicillin and streptomycin, Sigma-Aldrich, St. Louis, MO, USA) under standard culture conditions at 37 °C in a humidified atmosphere containing 5% CO_2_. When needed, the cells were rinsed with phosphate-buffered saline (PBS, Sigma-Aldrich, St. Louis, MO, USA) and removed by trypsinization (0.025% trypsin and 0.02% EDTA; Sigma-Aldrich, St. Louis, MO, USA).

### 4.2. Quantification of the Total Cholesterol Content

We quantified the total cholesterol content with Cholesterol/Cholesteryl Ester Quantitation Assay Kit (Abcam, ab65359, Cambridge, UK). To assess the total cholesterol content in the cells preincubated with 50 nM atorvastatin, 1 × 10^6^ cells were seeded on cover glasses in Petri dishes for 48 h. Afterward, the cells were washed 3 times with PBS and further suspended in chloroform. Then, the cells were homogenized with the microhomogenizer and resuspended in PBS. Following the manufacturer’s protocol, the samples were centrifugated and chloroform was evaporated. The dry sample was resuspended in the cholesterol buffer and the solution was moved to the 96-well plate. The cholesterol esterase was added into each well to convert the cholesterol esters to free cholesterol. After 30 min of incubation, the reaction mixture was added and incubated for 30 min. The results were gathered on the spectrophotometer (492 nm). Simultaneously with the experiment, the standard cholesterol absorption curve was prepared. The value of cholesterol mass in the experimental probes was evaluated with the standard cholesterol curve.

### 4.3. Confocal Microscopy Studies of Cholesterol Distribution

Confocal microscopy was used to visualize the cholesterol distribution in the A375, MDA-MB231, and DU 145 cells. We performed staining of the steroid according to the Cholesterol Assay Kit (Abcam, ab133116, UK). For each cell line, one sample was incubated with 50 nM atorvastatin and the second one was the control. The cells were incubated for 48 h, and afterward, the cells were washed with PBS and further incubated for 1 h with the medium containing flipin-2. After the incubation, the medium was removed and the cells were washed with PBS. Then, the samples were observed under the confocal scanning laser microscope Olympus FluoView FV1000 (Olympus, Tokyo, Japan). An oil immersion lens with 60× magnification, NA: 1.35 (Olympus, Tokyo, Japan) was used to capture the images. Photographs were taken to assess cholesterol distribution in the cells with and without preincubation with 50 nM atorvastatin.

### 4.4. Fluorescent Staining of Actin and Zyxin

To visualize the actin filaments and zyxin in the cells, fluorescence microscopy was used. The cells were incubated on cover glasses in Petri dishes for 48 h with 50 nM atorvastatin. Control samples were prepared as well. Afterward, the cells were washed 3 times with PBS. Actin filaments were stained with Invitrogen™ Alexa Fluor™ 546 Phalloidin (2 μg/mL, Thermo Fisher Scientific, A22283, Waltham, MA, USA) with the producer’s standard protocol. Zyxin was targeted with primary antibody (MAB6977, RD systems, Minneapolis, MN, USA) at 3 µg/mL followed by staining with a mixture of Alexa Fluor 488 dye (2 μg/mL, A11029, Thermo Fisher Scientific, Waltham, MA, USA). Samples were observed with the confocal scanning laser microscope Olympus FluoView FV1000 (Olympus, Tokyo, Japan). An oil immersion lens with 60× magnification, NA: 1.35 (Olympus, Tokyo, Japan) was used to capture the images.

### 4.5. PEF Experiment

The culture medium was removed by centrifugation and the cells were suspended in the electroporation buffer (10 mM HEPES buffer pH = 7.4, 1 mM MgCl_2_ and 250 mM sucrose). The square wave electroporator was used to deliver the electric pulses (BTX, Syngen Biotech, Poland, Wroclaw) and cuvettes with a 1 mm distance between the electrodes (BTX, Harvard, MA, USA) with or without 5 mM calcium chloride. Electric fields ranging from 0 to 1200 V/cm were applied. Each experiment involved the application of 8 pulses with 1 Hz frequency (0.1 ms pulse duration). After pulsed electric fields, cells were left for 1 min at 37 °C in a humidified atmosphere containing 5% of CO_2_. After this time, cells were prepared for further analysis according to the procedure.

For SRB and MTT assay, cells were seeded into 96-well microculture plates at a concentration of 2 × 10^4^ cells/well incubated for 48 h, 10^4^ cells/well incubated for 72 h, and 5 × 10^3^ cells/well incubated for 120 h at 37 °C 5% CO_2_.

### 4.6. Yo-Pro-1 Uptake—Flow Cytometry Studies

For detection of cell membrane permeabilization, Yo-Pro-1 (1:1000, Y3603, Thermo Fisher Scientific, Waltham, MA, USA) was added to each sample before electroporation, following by flow cytometric analysis. Yo-Pro-1 dye is commonly used in permeabilization studies due to the fact that it only flows into permeabilized or necrotic cells. Unstained and stained negative as well as positive controls were included in the experiments. Afterward, the cells were treated with electric fields ranging from 500 to 1200 V/cm with 100 V/cm step (8 pulses, 1 Hz, 0.1 ms pulse duration). Flow cytometry analysis was performed using a Cube 6 flow cytometer (Sysmex, Warsaw, Poland). The fluorescence of Yo-Pro-1 was excited with 488 nm laser and assessed with the FL-1-detector (525/50). Data were collected and analyzed by CyView software (Sysmex, Warsaw, Poland).

### 4.7. Impact of Cholesterol on the Electroporation Threshold—MD Study

The model membrane was composed of ~256 lipids per leaflet solvated in a NaCl solution and built using the CHARMM-GUI web interface and visually inspected with VMD software [50,51]. All the systems were built considering mixture of lipids-cholesterol and 1-palmitoyl-2-oleoyl-sn-glycero-3-phosphocholine–POPC. Four systems representing 10%, 30%, 50%, and 70% cholesterol were modeled, each with POPC. Six calcium ions were considered in each system.

The simulations were performed using 3D periodic boundary conditions using the CHARMM36 force field [52] and the GROMACS 2020.5 software [53]. The systems were first minimized then equilibrated (100 ns) at constant pressure using the Nose-Hoover thermostat and Berendsen barostat. To subject the systems to a TM voltage, we used the single bilayer setup previously introduced by us [54,55]. Briefly, a void slab is created along the normal (*z*-axis) to the membrane in order to separate the ionic bath from their periodic images. Such a setup allows for adequately modeling the system submitted to a given transmembrane voltage. For each system considered, the simulations were conducted at increasing voltages to determine the electroporation threshold, i.e., the applied TM voltage at which pores form in the bilayer. We assessed the electroporation threshold for the bilayer where the surface tension was maintained at 0 mimicking a tensionless membrane, and for membranes with a positive and negative surface tensions of ~30 mN/m.

As for the analysis, the potential of the whole system was calculated on the trajectory of about 1000 steps before the electropore formed. The systems were centered and the potential was calculated. Potential plots were derived and afterwards the water slabs were moved to 0 TM. Afterwards, the new charge plot was integrated and from the obtained plot, we calculated the transmembrane voltage in the electroporation threshold point.

### 4.8. Viability Assays

During treatment with statins, the drug might have an immediate effect on the cells or induce changes that take longer times to manifest. To investigate the long- and short-term effect of the statin on the efficacy of the PEF treatments, we analyzed three types of samples: (1) samples of cells without atorvastatin; (2) samples of cells preincubated with 50 nM atorvastatin for 48 h before PEF treatment (long-term effects); and (3) samples of cells treated with 50 nM atorvastatin directly in the electroporation buffer, i.e., without pre-incubation time.

#### 4.8.1. SRB Assay

After delivering the electric pulses with and without 5 mM calcium chloride in HEPES buffer, 10^4^ cells per well were seeded into 96-well culture plates for 72 h incubation. After this time the medium was removed from the cells and 50 µL of 50% cold trichloroacetic acid (TCA, Sigma-Aldrich, St. Louis, MO, USA) was added to each well and incubated for 1 h at 4 °C. Afterward, the plate was washed with water and then dried. Then, 50 µL of 0.4% sulforhodamine B (SRB, Sigma-Aldrich, St. Louis, MO, USA) solution in 1% acetic acid (Sigma-Aldrich, St. Louis, MO, USA) was added to each well. Incubation was performed for 30 min at room temperature. Further, the plate was washed 5 times with 1% acetic acid (Sigma-Aldrich, St. Louis, MO, USA) and dried. Finally, 150 µL of 10 mM TRIS solution (Sigma-Aldrich, St. Louis, MO, USA) was added to each well. The absorbance reading at 492 nm wavelength was performed with a microplate reader (GloMax^®^ Discover, Promega, GmbH, Walldorf, Germany).

#### 4.8.2. MTT Mitochondrial Activity Assay

After delivering the electric pulses with and without 5 mM calcium chloride in HEPES buffer, cells were seeded into 96-well microculture plates at a concentration of 2 × 10^4^ cells/well incubated for 48 h, 5 × 10^3^ cells/well incubated for 120 h at 37 °C 5% CO_2_. After 48 or 120 h after the experiment, the culture medium was removed from the wells, and 100 μL of 0.5 mg/mL MTT (3-(4,5-dimethylthiazol-2-yl)-2,5-diphenyltetrazolium bromide, Sigma) solution in PBS buffer was added to the 96-well plates; for the 6-well plates, the volume of MTT reagent was 0.5 mL. After 2 h incubation at 37 °C, acidified isopropanol (100 μL, 0.04 M HCl in 99.9% isopropanol) was added to dissolve the formazan crystals. Each well’s absorbance was measured at 570 nm using the multiplate reader (Promega, GmbH, Germany). The results were normalized to control (100%) and plotted.

#### 4.8.3. Clonogenic Assay

To assess the colony-forming properties after the therapy, the cells were seeded in dilutions (1000 cells) on 6-well plates. Plates were placed in an incubator and left untouched for 10 days until colonies were observed in the control samples. After the incubation, DMEM was removed, and the cells were washed with PBS. Staining of clones was performed with a mixture of 0.5% crystal violet in 4% paraformaldehyde (PFA, Sigma-Aldrich, St. Louis, MO, USA) for 10 min. Afterward, the free stain was removed by washing with water and being left to dry at room temperature. Next, only the eye-visible colonies (>~0.02 cm) were counted manually. The counting of the colonies was unbiased—the counting person was not familiar with the samples’ IDs. Further, the data were plotted.

### 4.9. E- and N-Cadherin Immunofluorescence Staining Studies

Given the fact that statins alter cholesterol metabolism, various changes in the expression of raft-bound proteins might be expected in the studied cells’ plasma membranes upon exposition to atorvastatin. In order to examine the effects of cholesterol depletion on these membranes, we designed experiments to specifically determine the expression and localization of E- and N-cadherins. An increased fluorescent signal of N-cadherin in the cell membrane localization is associated with the cell’s mesenchymal phenotype and thus relates to the increase in the epithelial-derived cancer cells’ metastatic properties. Conversely, increased E-cadherin membrane expression is correlated with the epithelial cell morphology and lower metastatic potential. We analyzed the localization and expression of E- and N-cadherins on human cancer cell lines. However, not each cadherin is present on all cell lines, thus we stained only cells with the proven expression of each type of cadherin. MDA-MB231 were deficient for both cadherins [56,57]. A375 cells expressed only N-cadherin [58,59] and DU 145 cells were E-cadherin positive [60,61,62].

Confocal laser scanning microscopy was applied to assess E- and N-cadherins’ expression and distribution in the A375 and DU 145 cells after 5 mM calcium electroporation preceded by preincubation with 50 nM atorvastatin. MDA-MB231 were proven to lack the expression of E-cadherin, thus we left the cells for the study. The cells were incubated for 48 h with 50 nM atorvastatin. Then, the cells were detached and electroporated with and without 5 mM calcium chloride. Afterward, the cells were seeded on the coverslips in the Petri dish. After 48 h, the cells were fixed with 10% formalin and stained according to the manufacturers’ protocols. After fixation, the cells were permeabilized with Triton X-100 (5%) and incubated with BSA for 1 h in 37 °C. Afterward, the cells were rinsed with PBS-Triton-X-100 solution and the solution of the first-order antibody was added. For E-cadherin’s examination, the mouse monoclonal antibody (1:500; HECD-1, Thermo Fisher, Waltham, MA, USA) was used. For N-cadherin’s assessment, the rabbit monoclonal antibody (1:200; SY02-46, Thermo Fisher Scientific, Waltham, MA, USA) was used. After 1 h of incubation, the cells were washed with PBS solution and the secondary antibody was conjugated with Alexa Fluor-488 (4 μg/mL; A11008 for E-cadherin and A11029 for N-cadherin, Thermo Fisher Scientific, Waltham, MA, USA) was added and left for 0.75 h incubation in 37 °C. Following the incubation, the cells were washed in PBS. Fluorshield^TM^ was applied to mount the cells. The samples were observed on the Olympus FluoView FV1000 confocal laser scanning microscope (Olympus, Tokyo, Japan). An oil immersion lens with 60× magnification, NA: 1.35 (Olympus, Tokyo, Japan), was used to capture the images.

### 4.10. Statistical Analysis

The experiments were performed in biological replicates. Flow cytometry Yo-Pro uptake studies were performed in technical replicates. Data on the plots were expressed as mean ± SD and were analyzed by ANOVA coupled with a multiple comparison technique—Tukey’s test (in GraphPad Prism 8), with *p* < 0.05 being considered statistically significant (ns: not significant). The number of replicates (*n*) taken for the statistical analysis was presented below the figures.

## 5. Conclusions

To conclude, our viability experiments showed that the most efficient protocol of PEF treatment involves high voltage (1200 V/cm) PEFs in combination with supplementing calcium ions, namely CaEP. The results presented demonstrate that depending on the cell line, the combination with atorvastatin may be beneficial but only when high voltages are applied. Due to the differences between the three cell lines responses, atorvastatin should not be considered an enhancing factor for ECT.

Atorvastatin acts on the cells by reorganizing (disturbing) their cytoskeleton actin fibers and decreasing their cholesterol content. In response to the actin cytoskeleton reorganization, the electroporation threshold increases, presumably due to the cell’s higher resistance to the induction of the membrane’s tension. At higher intensity, the cholesterol content in the membrane plays a more significant role and steroidogenic cells with low actin response to atorvastatin preincubation are characterized by the higher permeability of the membrane after EP. These molecular changes affect the viability and colony forming properties of the cells when low voltage PEFs are applied, but when the PEF intensity increases to 1200 V/cm, the differences diminish. Thus, higher applied voltages imply a more consistent outcome of electroporation.

## Data Availability

The data presented in this study are available on request from the corresponding author.

## References

[1] Tieleman D.P. (2004). The molecular basis of electroporation. BMC Biochem..

[2] Kotnik T., Rems L., Tarek M., Miklavčič D. (2019). Membrane Electroporation and Electropermeabilization: Mechanisms and Models. Annu. Rev. Biophys..

[3] Kiełbik A., Szlasa W., Michel O., Szewczyk A., Tarek M., Saczko J., Kulbacka J. (2020). In Vitro Study of Calcium Microsecond Electroporation of Prostate Adenocarcinoma Cells. Molecules.

[4] Frandsen S.K., Vissing M., Gehl J. (2020). A comprehensive review of calcium electroporation—A novel cancer treatment modality. Cancers.

[5] Frandsen S.K., Gissel H., Hojman P., Tramm T., Eriksen J., Gehl J. (2012). Direct Therapeutic Applications of Calcium Electroporation to Effectively Induce Tumor Necrosis. Cancer Res..

[6] Kiełbik A., Szlasa W., Saczko J., Kulbacka J. (2020). Electroporation-Based Treatments in Urology. Cancers.

[7] Blazek A.D., Paleo B.J., Weisleder N. (2015). Plasma Membrane Repair: A Central Process for Maintaining Cellular Homeostasis. Physiology.

[8] Horn A., Jaiswal J.K. (2019). Structural and signaling role of lipids in plasma membrane repair. Current Topics in Membranes.

[9] Kandušer M., Šentjurc M., Miklavčič D. (2006). Cell membrane fluidity related to electroporation and resealing. Eur. Biophys. J..

[10] Saulis G. (1997). Pore disappearance in a cell after electroporation: Theoretical simulation and comparison with experiments. Biophys. J..

[11] Rembiałkowska N., Dubińska-Magiera M., Sikora A., Szlasa W., Szewczyk A., Czapor-Irzabek H., Daczewska M., Saczko J., Kulbacka J. (2020). Doxorubicin assisted by microsecond electroporation promotes irreparable morphological alternations in sensitive and resistant human breast adenocarcinoma cells. Appl. Sci..

[12] Hoejholt K.L., Mužić T., Jensen S.D., Dalgaard L.T., Bilgin M., Nylandsted J., Heimburg T., Frandsen S.K., Gehl J. (2019). Calcium electroporation and electrochemotherapy for cancer treatment: Importance of cell membrane composition investigated by lipidomics, calorimetry and in vitro efficacy. Sci. Rep..

[13] Mir L.M., Glass L.F., Sersa G., Teissie J., Domenge C., Miklavcic D., Jaroszeski M.J., Orlowski S., Reintgen D.S., Rudolf Z. (1998). Effective treatment of cutaneous and subcutaneous malignant tumours by electrochemotherapy. Br. J. Cancer.

[14] Marty M., Sersa G., Garbay J.R., Gehl J., Collins C.G., Snoj M., Billard V., Geertsen P.F., Larkin J.O., Miklavcic D. (2006). Electrochemotherapy-An easy, highly effective and safe treatment of cutaneous and subcutaneous metastases: Results of ESOPE (European Standard Operating Procedures of Electrochemotherapy) study. Eur. J. Cancer Suppl..

[15] Miklavčič D., Mali B., Kos B., Heller R., Serša G. (2014). Electrochemotherapy: From the drawing board into medical practice. Biomed. Eng. Online.

[16] Szlasa W., Zendran I., Zalesińska A., Tarek M., Kulbacka J. (2020). Lipid composition of the cancer cell membrane. J. Bioenerg. Biomembr..

[17] Raffy S., Teissie J. (1999). Control of Lipid Membrane Stability by Cholesterol Content. Biophys. J..

[18] Cooper R.A. (1979). Influence of increased membrane cholesterol on membrane fluidity and cell function in human red blood cells. Prog. Clin. Biol. Res..

[19] Huang B., Song B., Xu C. (2020). Cholesterol metabolism in cancer: Mechanisms and therapeutic opportunities. Nat. Metab..

[20] Zhang X., Barraza K.M., Beauchamp J.L. (2018). Cholesterol provides nonsacrificial protection of membrane lipids from chemical damage at air–water interface. Proc. Natl. Acad. Sci. USA.

[21] Rivel T., Ramseyer C., Yesylevskyy S. (2019). The asymmetry of plasma membranes and their cholesterol content influence the uptake of cisplatin. Sci. Rep..

[22] Muralidharan A., Rems L., Kreutzer M.T., Boukany P.E. (2021). Actin networks regulate the cell membrane permeability during electroporation. Biochim. Biophys. Acta-Biomembr..

[23] Ginsberg H.N. (2006). Review: Efficacy and mechanisms of action of statins in the treatment of diabetic dyslipidemia. J. Clin. Endocrinol. Metab..

[24] Stancu C., Sima A. (2001). Statins: Mechanism of action and effects. J. Cell. Mol. Med..

[25] Di Bello E., Zwergel C., Mai A., Valente S. (2020). The Innovative Potential of Statins in Cancer: New Targets for New Therapies. Front. Chem..

[26] Lennern H. (2003). Clinical Pharmacokinetics of Atorvastatin. Clin. Pharmacokinet..

[27] Bakker-Arkema R.G., Best J., Fayyad R., Heinonen T.M., Marais A.D., Nawrocki J.W., Black D.M. (1997). A brief review paper of the efficacy and safety of atorvastatin in early clinical trials. Atherosclerosis.

[28] Fan Z., Jiang H., Wang Z., Qu J. (2016). Atorvastatin partially inhibits the epithelial-mesenchymal transition in A549 cells induced by TGF-β1 by attenuating the upregulation of SphK1. Oncol. Rep..

[29] Rafehi H., Orlowski C., Georgiadis G.T., Ververis K., El-Osta A., Karagiannis T.C. (2011). Clonogenic assay: Adherent cells. J. Vis. Exp..

[30] Pichi B., Pellini R., de Virgilio A., Spriano G. (2018). Electrochemotherapy: A well-accepted palliative treatment by patients with head and neck tumours. Acta Otorhinolaryngol. Ital..

[31] Campana L.G., Edhemovic I., Soden D., Perrone A.M., Scarpa M., Campanacci L., Cemazar M., Valpione S., Miklavčič D., Mocellin S. (2019). Electrochemotherapy–Emerging applications technical advances, new indications, combined approaches, and multi-institutional collaboration. Eur. J. Surg. Oncol..

[32] De Virgilio A., Ralli M., Longo L., Mancini P., Attanasio G., Atturo F., De Vincentiis M., Greco A. (2018). Electrochemotherapy in head and neck cancer: A review of an emerging cancer treatment (Review). Oncol. Lett..

[33] Gehl J., Serša G. (2017). Electrochemotherapy and its clinical applications. Handbook of Electroporation.

[34] Tumwine L.K., Kagimu M., Ocama P., Segamwenge I., Masiira-Mukasa N., Wamala D., Dworak O., Opio C.K. (2012). Atypical presentation of colon adenocarcinoma: A case report. J. Med. Case Rep..

[35] Kiełbik A., Szlasa W., Novickij V., Szewczyk A., Maciejewska M., Saczko J., Kulbacka J. (2021). Effects of high-frequency nanosecond pulses on prostate cancer cells. Sci. Rep..

[36] Jose M.A., Anandkumar S., Narmadha M.P., Sandeep M. (2012). A comparative effect of atorvastatin with other statins in patients of hyperlipidemia. Indian J. Pharmacol..

[37] Ye Y.C., Zhao X.L., Zhang S.Y. (2015). Use of atorvastatin in lipid disorders and cardiovascular disease in chinese patients. Chin. Med. J. Engl..

[38] Sadeghi R., Asadpour-Piranfar M., Asadollahi M., Taherkhani M., Baseri F. (2014). The effects of different doses of atorvastatin on serum lipid profile, glycemic control, and liver enzymes in patients with ischemic cerebrovascular accident. ARYA Atheroscler..

[39] Adams S.P., Tsang M., Wright J.M. (2015). Lipid-lowering efficacy of atorvastatin. Cochrane Database Syst. Rev..

[40] Toth P.P., Banach M. (2019). Statins: Then and Now. Methodist Debakey Cardiovasc. J..

[41] Kakorin S., Liese T., Neumann E. (2003). Membrane curvature and high-field electroporation of lipid bilayer vesicles. J. Phys. Chem. B.

[42] Tarek M. (2005). Membrane Electroporation: A Molecular Dynamics Simulation. Biophys. J..

[43] Graybill P.M., Davalos R.V. (2020). Cytoskeletal Disruption after Electroporation and Its Significance to Pulsed Electric Field Therapies. Cancers.

[44] Casciola M., Bonhenry D., Liberti M., Apollonio F., Tarek M. (2014). A molecular dynamic study of cholesterol rich lipid membranes: Comparison of electroporation protocols. Bioelectrochemistry.

[45] Polak A., Tarek M., Tomšič M., Valant J., Ulrih N.P., Jamnik A., Kramar P., Miklavčič D. (2014). Electroporation of archaeal lipid membranes using MD simulations. Bioelectrochemistry.

[46] Polak A., Velikonja A., Kramar P., Tarek M., Miklavčič D. (2015). Electroporation threshold of POPC lipid bilayers with incorporated polyoxyethylene glycol (C12E8). J. Phys. Chem. B.

[47] Szlasa W., Supplitt S., Drąg-Zalesińska M., Przystupski D., Kotowski K., Szewczyk A., Kasperkiewicz P., Saczko J., Kulbacka J. (2020). Effects of curcumin based PDT on the viability and the organization of actin in melanotic (A375) and amelanotic melanoma (C32)– in vitro studies. Biomed. Pharmacother..

[48] Kim Y., Lee E.J., Jang H.K., Kim C.H., Kim D.-G., Han J.-H., Park S.M. (2016). Statin pretreatment inhibits the lipopolysaccharide-induced epithelial-mesenchymal transition via the downregulation of toll-like receptor 4 and nuclear factor-κB in human biliary epithelial cells. J. Gastroenterol. Hepatol..

[49] Warita K., Warita T., Beckwitt C.H., Schurdak M.E., Vazquez A., Wells A., Oltvai Z.N. (2014). Statin-induced mevalonate pathway inhibition attenuates the growth of mesenchymal-like cancer cells that lack functional E-cadherin mediated cell cohesion. Sci. Rep..

[50] Jo S., Kim T., Iyer V.G., Im W. (2008). CHARMM-GUI: A web-based graphical user interface for CHARMM. J. Comput. Chem..

[51] Humphrey W., Dalke A., Schulten K. (1996). VMD: Visual molecular dynamics. J. Mol. Graph..

[52] Vanommeslaeghe K., Hatcher E., Acharya C., Kundu S., Zhong S., Shim J., Darian E., Guvench O., Lopes P., Vorobyov I. (2010). CHARMM general force field: A force field for drug-like molecules compatible with the CHARMM all-atom additive biological force fields. J. Comput. Chem..

[53] Abraham M.J., Murtola T., Schulz R., Páll S., Smith J.C., Hess B., Lindah E. (2015). Gromacs: High performance molecular simulations through multi-level parallelism from laptops to supercomputers. SoftwareX.

[54] Casciola M., Kasimova M.A., Rems L., Zullino S., Apollonio F., Tarek M. (2016). Properties of lipid electropores I: Molecular dynamics simulations of stabilized pores by constant charge imbalance. Bioelectrochemistry.

[55] Casciola M., Tarek M. (2016). A molecular insight into the electro-transfer of small molecules through electropores driven by electric fields. Biochim. Biophys. Acta-Biomembr..

[56] Nieman M.T., Prudoff R.S., Johnson K.R., Wheelock M.J. (1999). N-cadherin promotes motility in human breast cancer cells regardless of their E-cadherin expression. J. Cell Biol..

[57] Hazan R.B., Kang L., Whooley B.P., Borgen P.I. (1997). N-Cadherin Promotes Adhesion between Invasive Breast Cancer Cells and the Stroma. Cell Commun. Adhes..

[58] Chung H., Jung H., Jho E.H., Multhaupt H.A.B., Couchman J.R., Oh E.S. (2018). Keratinocytes negatively regulate the N-cadherin levels of melanoma cells via contact-mediated calcium regulation. Biochem. Biophys. Res. Commun..

[59] Amschler K., Beyazpinar I., Erpenbeck L., Kruss S., Spatz J.P., Schön M.P. (2019). Morphological Plasticity of Human Melanoma Cells Is Determined by Nanoscopic Patterns of E- and N-Cadherin Interactions. J. Investig. Dermatol..

[60] Tran N.L., Nagle R.B., Cress A.E., Heimark R.L. (1999). N-cadherin expression in human prostate carcinoma cell lines: An epithelial-mesenchymal transformation mediating adhesion with stromal cells. Am. J. Pathol..

[61] Nalla A.K., Estes N., Patel J., Rao J.S. (2011). N-cadherin mediates angiogenesis by regulating monocyte chemoattractant protein-1 expression via PI3K/Akt signaling in prostate cancer cells. Exp. Cell Res..

[62] Wang M., Ren D., Guo W., Huang S., Wang Z., Li Q., Du H., Song L., Peng X. (2016). N-cadherin promotes epithelial-mesenchymal transition and cancer stem cell-like traits via ErbB signaling in prostate cancer cells. Int. J. Oncol..

